# Supplementary material for: Generation of human colon organoids from healthy and inflammatory bowel disease mucosa
Source: PLoS One. 2022 Oct 27;17(10):e0276195. doi: 10.1371/journal.pone.0276195 (PMC9612551; doi:10.1371/journal.pone.0276195)
Supplement: S1 File — Also available on protocols.io. (PDF) [file pone.0276195.s001.pdf]

cfh7tj9n

Generation of human colon organoids from healthy and inflammatory bowel disease mucosa V.(cfh7tj9n)

Isabella Dotti[1], Aida Mayorgas[1], Azucena Salas[1]

[1]Department of Gastroenterology, IDIBAPS, Hospital Clínic, CIBER-EHD, Barcelona, Spain

1 Works for me

Reserved DOI:
10.17504/protocols.io.rm7vz3wr4gx1/v2

Isabella Dotti

ABSTRACT

Ulcerative colitis and Crohn's Disease are chronic inflammatory bowel diseases (IBD) of unknown cause characterized by a relapsing-remitting behavior. Growing evidence supports the idea that the epithelial barrier plays a central role in the pathogenesis of IBD as well as in its evolution over time, thus representing a potential target for novel therapeutic options.
In the last decade, the introduction of 3D epithelial cultures from *ex vivo*-expanded intestinal adult stem cells (ASCs) has impacted our ability to study the function of the epithelium in several gastrointestinal disorders, including IBD.
Here, we describe in detail a reproducible protocol to generate Matrigel-embedded epithelial organoids from ASCs of non-IBD and IBD donors using small colonic biopsies, including steps for its optimization. A slightly modified version of this protocol is also provided in case surgical samples are used.
With this method, epithelial organoids can be expanded over several passages, thereby generating a large quantity of viable cells that can be used in multiple downstream analyses including genetic, transcriptional, proteomic and/or functional studies. In addition, 3D cultures generated using our protocol are suitable for the establishment of 2D cultures, which can model relevant cell-to-cell interactions that occur in IBD mucosa.

PROTOCOL INFO

Isabella Dotti, Aida Mayorgas, Azucena Salas . Generation of human colon organoids from healthy and inflammatory bowel disease mucosa . **protocols.io** https://protocols.io/view/generation-of-human-colon-organoids-from-healthy-a-cfh7tj9n
Version created by Isabella Dotti

FUNDERS ACKNOWLEDGEMENT

3TR
Grant ID: 831434

ECCO Grant

MANUSCRIPT CITATION please remember to cite the following publication along with this protocol

Dotti I, Mora-Buch R, Ferrer-Picón E, Planell N, Jung P, Masamunt MC, Leal RF, Martín de Carpi J, Llach J, Ordás I, Batlle E, Panés J, Salas A. Alterations in the epithelial stem cell compartment could contribute to permanent changes in the mucosa of patients with ulcerative colitis. Gut. 2017 Dec;66(12):2069-2079.

KEYWORDS

Adult stem cells, Inflammatory bowel diseases, Organoid culture, Human samples, Intestinal epithelium

IMAGE ATTRIBUTION

Image created by Alba Garrido-Trigo. All rights reserved.

CREATED

Aug 19, 2022

LAST MODIFIED

Aug 23, 2022

PROTOCOL INTEGER ID

68895

GUIDELINES

Approval from the ethics committee and signed consent by the donor must be obtained prior to human sample processing. In case of underage donors, the consent must be signed by the legal representatives.

MATERIALS TEXT

Biological material

- Fresh human colonic sample (4-8 biopsies or 1-2 cm^2 surgical samples) from non-IBD or IBD subject of pediatric or adult age. Informed consent must be obtained before sample processing.

Reagents

- Hank's Balanced Salt Solution (HBSS).
- Dulbecco's phosphate-buffered saline, without Ca^{2+} and Mg^{2+} (DPBS).
- Amphotericin B (ThermoFisher, Cat. No. 15290): aliquot and store at -20°C.
- Normocin (InvivoGen, Cat. No. ant-nr). Store at -20°C.
- Gentamicin (Lonza Bioscience, Cat. No. 17-519Z). Store at room temperature.
- Ethylenediaminetetraacetic acid (EDTA) solution, 0.5 M (pH 8). Store at room temperature.
- Dithiothreitol (DTT): prepare a 1M solution in distilled H_2O, aliquot and store at -20°C.
- Fetal bovine serum (FBS). Store at -20°C (see the following **Note**).

Inactivate FBS at 56°C for 30 min, and prepare 15-30 mL aliquots. Store the aliquots for 1-2 years maximum at -20°C. Once thawed, store the aliquot at 4°C for a maximum of 10 days. As organoid growth can be significantly affected by components of FBS, we recommend using the same lot of FBS throughout the

project to avoid experimental bias resulting from batch-to-batch variations.

- Matrigel basement membrane matrix, growth-factor reduced, phenol red-free (Corning, Cat. No. 356231). Store at -20°C/–80°C (see the following **Note**).

To prepare aliquots from a new Matrigel batch, thaw the bottle overnight on ice in a cold room, and prepare small aliquots using a cold 5 mL pipette and 1.5 mL Eppendorf tubes pre-chilled on ice. Frozen aliquots can be stored at -20°C/-80°C for 1 year. Once thawed, aliquots can be kept at 4°C for 7 days maximum. For establishing organoid cultures, we use undiluted Matrigel at a protein concentration between 8.5 and 10.5 mg/mL. Since Matrigel shows batch-to-batch variations due to its animal origin, we recommend first testing the efficiency of one bottle whenever a new batch is purchased, and then using the same batch throughout the entire project, if possible.

- Advanced DMEM/F12 (ThermoFisher, Cat No. 12634). Store at 4°C.
- Wnt3a-conditioned medium (Wnt3a-CM). Store at -20°C (see the following **Note**).

Wnt3a-CM is prepared using the mouse L Wnt-3A cell line (ATCC, CRL-2647). Briefly, expand an L Wnt-3A vial in a T75 flask containing DMEM + 10% FBS. Split cells every two days, or when they are 100% confluent. Perform three passages: the first two at a dilution of 1:3, and the third at 1:5.
Collect medium from the third passage, 48 and 96 hours later. Aliquot the CM in 50 mL Falcon tubes, spin at 3000 x rpm for 10 min at 4°C, and filter through a 0.22 µM filter. To assess the quality of the CM, test the batches in the organoid culture over 2-3 passages using the previously produced CM batch, if available, as a reference. Alternatively, the TOP/FOP assay can be used for testing the CM quality. If high-quality CM is produced, it should be divided into aliquots and stored at -20°C for a maximum one year. Once thawed, the aliquots can be stored at 4°C for a maximum of 10 days. The possibility of batch-to-batch variability should always be kept in mind; for this reason we recommend, whenever possible, using the same batch throughout the entire project.

- GlutaMAX (ThermoFisher, Cat. No. 35050). Store at 4°C.
- HEPES (ThermoFisher, Cat. No. 15630). Store at 4°C.
- N-2 Supplement (ThermoFisher, Cat. No. 17502048): aliquot and store at -20°C.
- B-27 Supplement without retinoic acid (ThermoFisher, Cat. No. 12587010): aliquot and store at -20°C.
- Nicotinamide (NAM; Sigma-Aldrich, Cat. No. NO636): prepare a 1M solution in distilled H_2O, aliquot and store at -20°C.
- N-Acetyl-L cysteine (NAC; Sigma-Aldrich, Cat. No. A9165): prepare a 500 mM solution in distilled H_2O, aliquot and store at -20°C.
- Human R-spondin-1 (RSPO1; Sino Biologicals, Cat. No. 11083-H08H): prepare a 250-500 µg/mL solution in distilled H_2O, aliquot and store at -80°C.

- Human Epidermal Growth Factor Recombinant Protein (EGF; ThermoFisher, Cat. No. PHG0311): prepare a 50 µg/mL solution in 0.1% BSA in DPBS, aliquot and store at -20°C.
- Human Noggin (Peprotech, Cat. No. 120-10C): prepare a 100-200 µg/mL solution in distilled H_2O, aliquot and store at -20°C.
- Human [Leu^{15}]-Gastrin I (Sigma-Aldrich, Cat. No. G9145): prepare a 100 µM solution in 0.1% BSA in DPBS, aliquot and store at -20°C.
- LY 2157299 (Axon MedChem, Cat. No. 1491): prepare a 25 mM solution in DMSO, aliquot and store at -80°C.
- SB 202190 (Sigma-Aldrich, Cat. No. S7067): prepare a 30 mM solution in DMSO, aliquot and store at -80°C.
- Prostaglandin E_2 (PGE_2; Sigma-Aldrich, Cat. No. P0409): dissolve the powder to 28 mM in 100% EtOH, prepare 2.5 mM and 50 µM solutions in 100 mM NaH_2PO_4, aliquot and store at -20°C.
- Y-27632 (Sigma-Aldrich, Cat. No. 688000): prepare a 5 mM solution in distilled H_2O, aliquot and store at -20°C.
- Cell recovery solution (Corning, Cat. No. 354253). Store at 4°C. Use ice cold.
- Dispase (ThermoFisher, Cat. No. 17105041): prepare a 40 mg/mL solution in DPBS, aliquot and store at -20°C.
- Recovery Cell Culture Freezing medium (ThermoFisher, Cat. No. 12648-010): Prepare 1 mL aliquots and store at -20°C.

The mouse L-WRN cell line (ATCC, CRL-3276) can be used as a source of Wnt3a, RSPO3 and noggin secreted into a single conditioned medium. In that case, the use of the recombinant proteins RSPO1 and noggin is not necessary. See the following publication for more information:

VanDussen KL, et al. L-WRN conditioned medium for gastrointestinal epithelial stem cell culture shows replicable batch-to-batch activity levels across multiple research teams. Stem Cell Res. 2019 May;37:101430.

Solution setup

- *Antimicrobial cocktail*: 2.5 µg/mL Amphotericin B, 500 µg/mL Normocin and 500 µg/mL gentamicin in DPBS, freshly prepared. Use at room temperature.
- *Crypt isolation buffer:* 8 mM EDTA and 500 µM DTT in cold DPBS for biopsy samples; 8 mM EDTA in cold DPBS for surgical samples. Freshly prepared. Use at 4°C.
- *Washing medium*: Advanced DMEM/F12 supplemented with 1x GlutaMAX, 10 mM HEPES and 5% FBS. Use ice cold. Store for up to 10 days at 4°C.
- *Basal medium*: Advanced DMEM/F12 supplemented with 1x GlutaMAX, 10 mM HEPES, 1x N-2 and 1x B-27. Use at room temperature. Store for up to 10 days at 4°C.
- *Organoid dissociation solution*: Advanced DMEM/F12 supplemented with 1x GlutaMAX, 10 mM HEPES, 1x N-2, 1x B-27, 10 mM NAM, 1mM NAC, 2.5 µM PGE_2, 10 µM Y-27632 and 400 µg/mL dispase. Freshly prepared. Use at room temperature.
- *Organoid growth medium*: Advanced DMEM/F12 and WNT3a-CM at 50% (v/v), supplemented with 1x GlutaMAX, 10 mM HEPES, 1x N-2, 1x B-27, 10mM NAM, 1mM NAC, 100 µg/mL Normocin, 500 ng/mL RSPO1, 50 ng/mL EGF, 100 ng/mL Noggin, 10 nM [Leu^{15}]-Gastrin I, 500 nM LY 2157299, 10 µM SB 202190, and 100 nM PGE_2. Use at room temperature. Store

for up to 10 days at 4°C.

- *Organoid differentiation medium*: Advanced DMEM/F12 supplemented with 1x GlutaMAX, 10mM HEPES, 1x N-2, 1x B-27 , 1 mM NAC, 100 µg/mL Normocin, 250 ng/mL RSPO1, 50 ng/mL EGF, 100 ng/mL Noggin, 10 nM [Leu^{15}]-Gastrin I and 500 nM LY 2157299. Use at room temperature. Store for up to 10 days at 4°C.

Once thawed, aliquots of reagents used for organoid dissociation solution and growth/differentiation media preparation can be stored at 4°C for a maximum of 2 weeks. Avoid repeated freeze-thaw cycles.

Equipment

- Autoclaved forceps, scissors, and scalpels.
- G20-G21 needles mounted on a 5 mL syringe.
- 48-well plates, flat bottom.
- 1.5 mL Eppendorf tubes.
- 15 mL and 50 mL Falcon tubes.
- Cryopreservation tubes.
- 10 cm Petri dishes.
- 5 mL, 10 mL and 25mL pipettes.
- Standard set of micropipettes.
- Platform rocker.
- Refrigerated benchtop centrifuge (with rotor for 15 mL and 50 mL tubes).
- Refrigerated microcentrifuge (with rotor for 1.5 mL tubes).
- Bright-field microscope.
- Water bath at 37°C.
- Freezing container.

1. Intestinal crypt isolation (day 1)

1 Collect 4-8 colonic biopsies (from non-IBD or IBD subject) obtained during the colonoscopy procedure, in a 15 mL Falcon tube containing 10 mL HBSS, preferably cold.

Immediate processing is highly recommended to prevent cell death. If this is not possible, keep the sample at 4°C for a maximum of 5-6 hours before proceeding with the protocol.

During intestinal crypt isolation (**Steps 1-13**), working under a sterile hood is not necessary.

ALTERNATIVE PROTOCOL: For isolation of intestinal crypts from a surgical sample, follow Steps 14-31

2 In this protocol, crypts are embedded in 25 µL-Matrigel drops seeded in a 48-well plate, one drop per well.

Based on the estimated number of crypts of the experiment (as determined in **Steps 32-33**), place the required number of Matrigel aliquots on ice for 1-2 hours before use, pre-warm the required number of 48-well plates in the cell incubator (5% CO_2, 37°C) about 1-2 hours before use, and prepare the required volume of *organoid growth medium*.

> The volume of the drops can be scaled according to the plate used. For example, 10 µL Matrigel drops can be plated in a well of a 96-well plate, or 4 drops of 25 µL can be plated in a well of a 12-well plate. We cannot guarantee that the integrity of the drop is maintained when higher volumes of Matrigel are used.

3 Wash the biopsies 1-2 times with ≈ 10 mL cold DPBS by gently flipping the tube for a few seconds.

4 Incubate the sample in 10 mL of the *antimicrobial cocktail* for 15 min at room temperature under gentle agitation on a platform rocker.

5 Wash 1-2 times with ≈ 10 mL DPBS at room temperature by gentle manual flipping for a few seconds to remove the *antimicrobial cocktail*.

6 Incubate the biopsies with 10 mL of the *crypt isolation buffer* for 45 min at 4°C under gentle agitation on a platform rocker.

7 Wash once with ≈ 10 mL cold DPBS by gentle manual flipping for a few seconds to remove the *crypt isolation buffer.*

8 To start collecting the fractions enriched with viable crypts, add 10 mL cold DPBS and shake vigorously by hand for 15-20 seconds. Depending on the sample, isolated crypts released from the tissue can be visible in the suspension in this first wash. Regardless, proceed to the next step.

9 Leave the biopsies to settle at the bottom of the tube and collect about 10 mL of the suspension in a new cold 15 mL Falcon tube using a 5 mL pipette. Add FBS to the suspension to a 5% final concentration to maintain cell viability.

Try to collect as many crypts as possible from each fraction to avoid the possibility that residual crypt are unnecessarily submitted to additional shakes, which could impair their quality.

10 Repeat **Steps 8-9** until no additional crypts are released. Usually, no crypts are collected after 5-6 shaking cycles.

11 Spin the crypt fractions at 200 x g for 3 min at 4°C.

12 Carefully remove the supernatant from each fraction using a 5 mL pipette and gently resuspend each crypt pellet in 1 mL cold *washing medium*. Merge the fractions in one 15 mL Falcon tube and spin again at 200 x g for 3 min at 4°C.

13 Resuspend the crypt pellet in cold w*ashing medium*. 1-2 mL *washing medium* are usually enough for an optimal crypt count. At this point, jump to **Step 32** or **Step 33** depending if crypt count is desired or not.

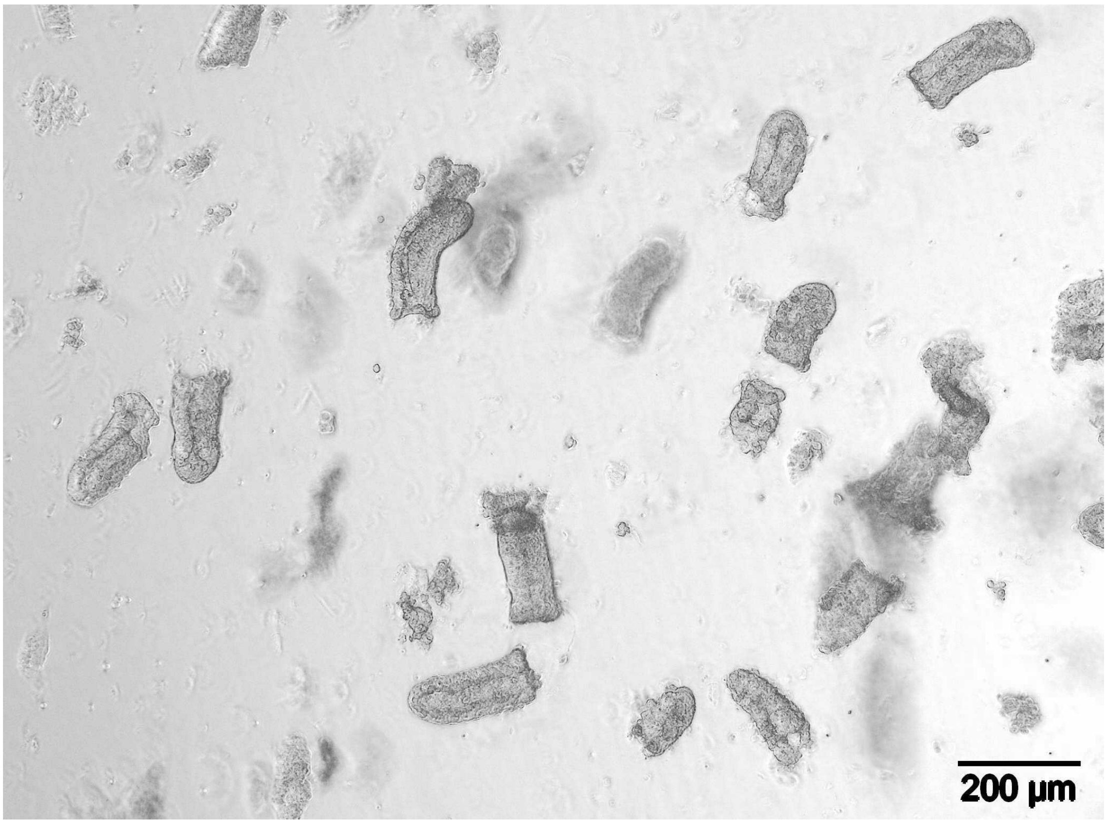

Crypts isolated from biopsies of a non-inflamed sigmoid mucosa of a CD patient.

Step 13 includes a Step case.
ALTERNATIVE PROTOCOL

step case

ALTERNATIVE PROTOCOL

This protocol is suitable for generating organoids mostly from non-IBD surgical samples , since in surgical samples from IBD patients the excessive inflammation can dramatically limit the isolation of viable crypts. This protocol is similar to the one used for biopsies, with some slight modifications. No extra reagents are required.

14 - Collect the fresh colonic surgical sample (1-2 cm^2-size) in a 50 mL Falcon tube containing ≈ 20 mL HBSS, preferably cold.

> Immediate processing is highly recommended to prevent cell death. If this is not possible, keep the sample at 4°C for a maximum of 5-6 hours before proceeding with the protocol.

> During crypt isolation (**Steps 14-31**), working under a sterile hood is not necessary.

15 In this protocol, crypts are embedded in 25 µL-Matrigel drops seeded in a 48-well plate, one drop per well.

Based on the estimated number of crypts of the experiment (as determined in **Steps 32-33**), place the required number of Matrigel aliquots on ice for 1-2 hours before use, pre-warm the required number of 48-well plates in the cell incubator (5% CO_2, 37°C) about 1-2 hours before use, and prepare the required volume of *organoid growth medium*.

> The volume of the drops can be scaled according to the plate used. For example, 10 µL Matrigel drops can be plated in a well of a 96-well plate, or 4 drops of 25 µL can be plated in a well of a 12-well plate. We cannot guarantee that the integrity of the drop is maintained when higher volumes of Matrigel are used.

16 Wash the surgical sample with ≈ 20 mL cold DPBS by gentle manual flipping for a few seconds. Discard the DPBS and repeat the washing until the DPBS becomes clear.

17 Transfer the sample to a 10 cm Petri dish and remove the submucosa and larger contaminating blood vessels with scissors and scalpel.

18 Cut the sample into pieces of 0.2-0.3 cm^2, transfer them to a new 50 mL Falcon tube and perform a couple of additional washes with ≈ 20 mL cold DPBS by gentle manual flipping for a few seconds.

> Avoid processing more than 15-20 sample pieces in one 50 mL Falcon tube since it could compromise the efficiency of crypt release and increase the risk of crypt culture contamination by microorganisms.

19 Incubate the sample in 20 mL of the *antimicrobial cocktail* for 20 min at room temperature under gentle agitation on a platform rocker.

20 Wash 1-2 times with ≈ 20 mL DPBS at room temperature to remove the *antimicrobial cocktail*.

21 Incubate with 10 mM DTT for 8 min at room temperature under gentle agitation on a platform rocker to remove the mucus.

22 Wash 1-2 times with ≈ 20 mL cold DPBS to remove DTT.

23 Incubate the sample pieces with 20 mL of *crypt isolation buffer* for 45 min at 4°C under gentle agitation on a platform rocker.

24 Remove the EDTA buffer and wash once with 20 mL cold DPBS by gentle flipping.

25 Resuspend the sample pieces in 20 mL cold DPBS and perform 1-2 manual vigorous shakes for 10-15 seconds each to remove the mucus, debris and dead cells. Usually, these supernatants are discarded since no viable crypts are detectable at this point.

26 To start collecting the fractions enriched with viable crypts, add 20 mL cold DPBS and shake vigorously by hand for 15-20 seconds. Crypts released from the tissue will be detectable in the suspension to the naked eye.

27 After each shake cycle, leave the sample pieces to settle at the bottom of the tube and collect about 20 mL of the suspension enriched in crypts in a new cold 50 mL Falcon tube using a 10 mL pipette. Add FBS to a 5% final concentration to maintain cell viability.

> Try to collect as many crypts as possible from each fraction to avoid the possibility that residual crypt are unnecessarily submitted to additional shakes, which could impair their quality.

28 Repeat **Steps 26-27** until the desired number of crypts is obtained.

> From a surgical sample large amounts of crypts can be released at each shaking cycle. Stop shaking when it is estimated that the desired number of crypts is obtained (refer to **Steps 32-33** for crypt counting).

29 Spin the crypt fractions at 150 x g for 3 min at 4°C.

30 Carefully remove the supernatant from each fraction using a 10 mL pipette and gently resuspend each crypt pellet in 1 mL cold *washing medium.* Merge the fractions in one 50 mL Falcon tube and spin again at 150 x g for 3 min at 4°C.

31 Resuspend the crypt pellet in cold *washing medium*. Depending on the size of the obtained crypt pellet, between 5- and 10-mL *washing medium* are usually necessary for an optimal crypt count.

2. Intestinal crypt culture (day 1 to day 3)

32 Place two or three 50 µL-drops of the crypt suspension on a glass slide to check crypt integrity and estimate the average number of crypts in the sample by bright-field microscopy examination.

> As a rule, when counting crypts, also include broken ones when the base is present: these will probably give rise to organoids since they still contain a viable stem cell compartment.

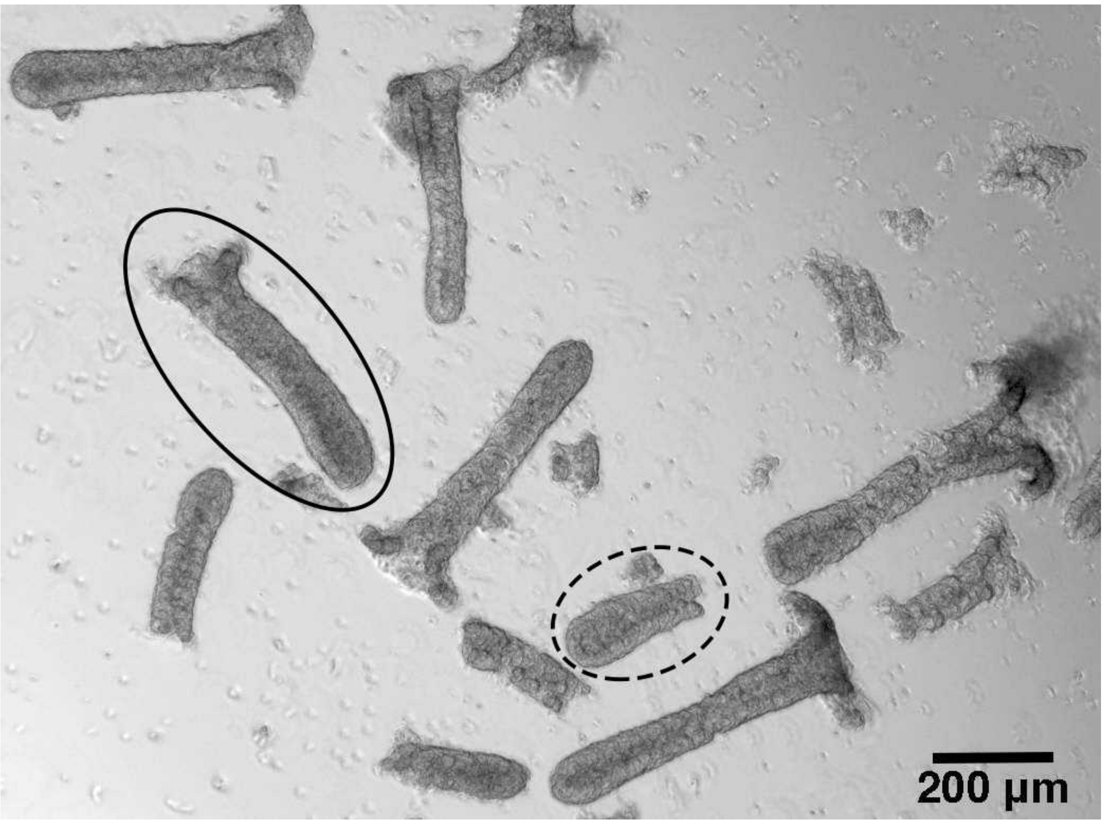

Example of a crypt suspension where both intact (circle) and broken (dotted circle) crypts are present. In this example, crypts were isolated from the surgical sample of a non-IBD sigmoid mucosa.

33 Take out the volume of crypts required for the planned experiment and transfer it to a new tube. We suggest embedding 80-100 crypts/25 µL-Matrigel drop seeded in a 48-well plate.

Since the amount of crypts isolated from biopsy samples is limited, **Step 32** may be skipped when using this type of specimen. Based on our experience, indeed, we calculate that on average 12-16 25µL-Matrigel drops are seeded in a 48-well plate when starting with 6-8 biopsies.

33.1 At this point the isolated crypts can be also used to prepare a short-term crypt culture. In this case we recommend using a lower number of crypts per Matrigel drop (about 50 crypts/25µl-drop). Refer to the following publication as an example of overnight crypt culture:

Calderón-Gómez E, et al. Commensal-Specific CD4(+) Cells From Patients With Crohn's Disease Have a T-Helper 17 Inflammatory Profile. Gastroenterology. 2016 Sep;151(3):489-500.e3.

34 Wash the tube containing the crypts with *washing medium* 2 times at 150-200 x g for 3 min at 4°C. Perform the second and last wash in a 1.5 mL Eppendorf tube if the required final volume of Matrigel fits in. Carefully aspirate the supernatant from the crypt pellet using a p1000 pipette.

35 Carefully resuspend the crypt pellet in the proper volume of ice-cold Matrigel in accordance with the number of crypt-Matrigel drops defined in the experimental plan. Avoid bubble formation.

From this point on, work under sterile conditions.

36 Plate a 25 µL crypt-Matrigel drop per well in a 48-well plate (or adjust depending on the well size) and return the plate to the incubator for 15-20 min to allow Matrigel solidification.

37 Overlay each Matrigel dome with 250 µL (or an appropriate volume depending on the well size) *organoid growth medium*. The crypt culture is usually ready to be expanded after 2-3 days, thus no medium change is usually required.

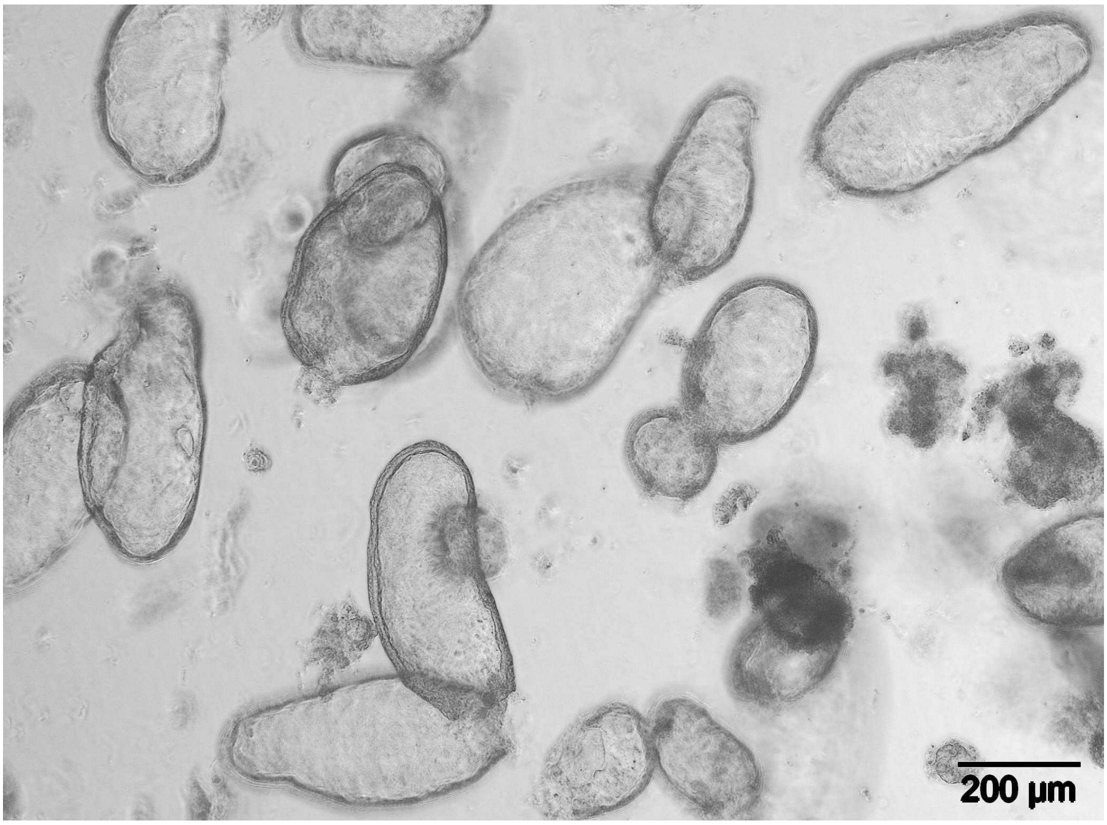

Crypt culture from the non-inflamed sigmoid mucosa of a CD patient 2 days after seeding.

3. Crypt/organoid expansion (day 3 to day 8)

38 ⚠

This protocol is suitable for expanding crypt cultures as well as previously

passaged organoid cultures.

Observe the crypt/organoid culture under the microscope to define the optimal dilution factor for expansion. Volumes indicated here are adjusted to 48 well-plates.

> A passage is usually performed 2-3 days after crypt seeding or every 5-6 days if the organoid culture has been previously expanded. As a rule, the dilution rate is 1:2-1:3 for a crypt culture, and 1:4-1:6 for previously expanded organoid cultures. Either way, dilution should be experimentally adjusted according to the estimated growth rate of the crypt/organoid culture.

39 Thaw the required number of Matrigel aliquots by placing them on ice 1-2 hours before use. Pre-warm the required number of 48-well plates in a cell incubator (5% CO_2, 37°C) for about 2-3 hours before use. Prepare *organoid growth medium* and warm it at room temperature before use. Work with ice-cold tubes and refrigerated centrifuges. Perform all the steps in a sterile biosafety cabinet.

40 Remove the *organoid growth medium* and wash the wells once with ≈ 250 µL cold DPBS per well.

41 Add 1 mL cold Recovery solution for every ≈ 3 drops of 25 µL Matrigel. Scrape the drops from the well surface with the P1000 micropipette tip and collect them in ice-cold 15 mL Falcon tubes. Avoid excessive pipetting since the organoids can stick to the tip and tube walls. Fill one Falcon tube with approximately 20 Matrigel drops maximum.

42 Incubate for 30-40 min on ice, gently inverting the tubes every 5-10 min to facilitate Matrigel depolymerization.

43 During **Step 42**, prepare the *organoid dissociation solution* (without yet adding the dispase to prevent loss of enzymatic activity) and pre-warm it in a water bath at 37°C. Consider that 5 mL solution will be prepared per Falcon tube containing the organoids/Recovery solution mix.

44 Fill each tube containing the Recovery solution and organoids with additional 5-7 mL cold WM and spin at 400 x g for 3 min at 4°C.

45 During the spin, add dispase to the *organoid dissociation solution*.

46 Discard the supernatant with a 5 mL pipette first, and then with a P1000 micropipette to

completely remove the solution. To resuspend the organoid pellet, first gently add 1 mL of the pre-warmed *organoid dissociation solution*, pipet gently 2-3 times, and then add the remaining 4 mL.

47 Leave the Falcon tubes in the cell incubator or in a water bath at 37°C for 15-20 min gently inverting the tubes every 5-10 min.

48 Mechanically dissociate the organoids with a G20-G21 needle mounted on a 5 mL syringe. It will take 2-5 min per tube, depending on weather the culture is derived from crypts or from a previously expanded organoid culture. Avoid generating too many bubbles in the solution while syringing.

> Organoid dissociation usually takes longer if performed on a previously expanded organoid culture than on a crypt culture. In either case, if big clusters are still present, repeat the syringing until no cell aggregates are visible in the suspension. The extent of organoid dissociation can also be monitored by microscope observation.

49 Spin dissociated organoids at 600-800 x g for 4 min at 4°C.

50 Discard the supernatant with a 5 mL pipette first, and then with a P1000 micropipette to completely remove the solution.

51 Perform 3 additional washes with ≈ 5 mL cold *washing medium* at 800 x g for 4 min at 4°C to completely remove the dispase. Perform the first two washes in the 15 mL Falcon tube and the last wash in a 1.5 mL Eppendorf tube if the required final volume of Matrigel fits in. At this point single cells from dissociated organoids can be cryopreserved (**Step 51.1**) or used for the generation of 2D cultures (**Step 51.2**).

51.1 For cryopreservation, perform the following:
- Transfer a volume of single cells corresponding to 4-6 original Matrigel drops to a new cold Eppedorf tube.
- Spin at 600-800 x g for 4 min at 4°C.
- Resuspend the cell pellet in 1 mL ice-cold Freezing Medium, transfer it to an ice-cold cryopreservation tube and move the sample to a freezing container.
- Store the sample at -80°C for 24h and transfer it to a liquid nitrogen container for long-term storage.
- To thaw, resuspend the cell-containing cryopreservation tube in 5-10 mL warm *washing medium.*
- Spin at 700 x g for 4 min at 4°C, resuspend in 1 mL cold *washing medium* and transfer it to a new cold Eppendorf tube.

- Spin again at 700 x g for 4 min at 4°C.
- At this point, go to **Step 52**.

For cryopreserved cultures, the suggested seeding dilution after thawing is lower than the one used for passaging fresh cultures. We usually work with a dilution ratio of 1:2-1:3.

51.2 For the establishment of a 2D culture, refer for example to the following publication describing in detail how to set a monolayer on conventional culture plates using organoid cultures generated by the proposed protocol:

Mayorgas A, et al. A Novel Strategy to Study the Invasive Capability of Adherent-Invasive *Escherichia coli* by Using Human Primary Organoid-Derived Epithelial Monolayers. Front Immunol. 2021 Mar 29;12:646906.

52 Resuspend the cell pellet in Matrigel supplemented with 10 µM Y-27632. The volume of Matrigel must be adjusted to the total number of drops (22-25µL Matrigel/drop) that will be seeded. Avoid bubbles while resuspending.

53 Seed the required Matrigel drops in the pre-warmed 48-well plate and put the plate back in the incubator for 15-20 min to allow the Matrigel to solidify.

54 Overlay each Matrigel-embedded dome with 250 µL of *organoid growth medium* supplemented with 10 µM Y-27632.

55 Change the medium with fresh *organoid growth medium* (without Y-27632) every 48-72h hours. Organoid cultures grown for 5-6 days are typically ready to be used for differentiation (**Steps 56-59**) or for further expansion by repeating the procedure described in this section (**Steps 38-55**).

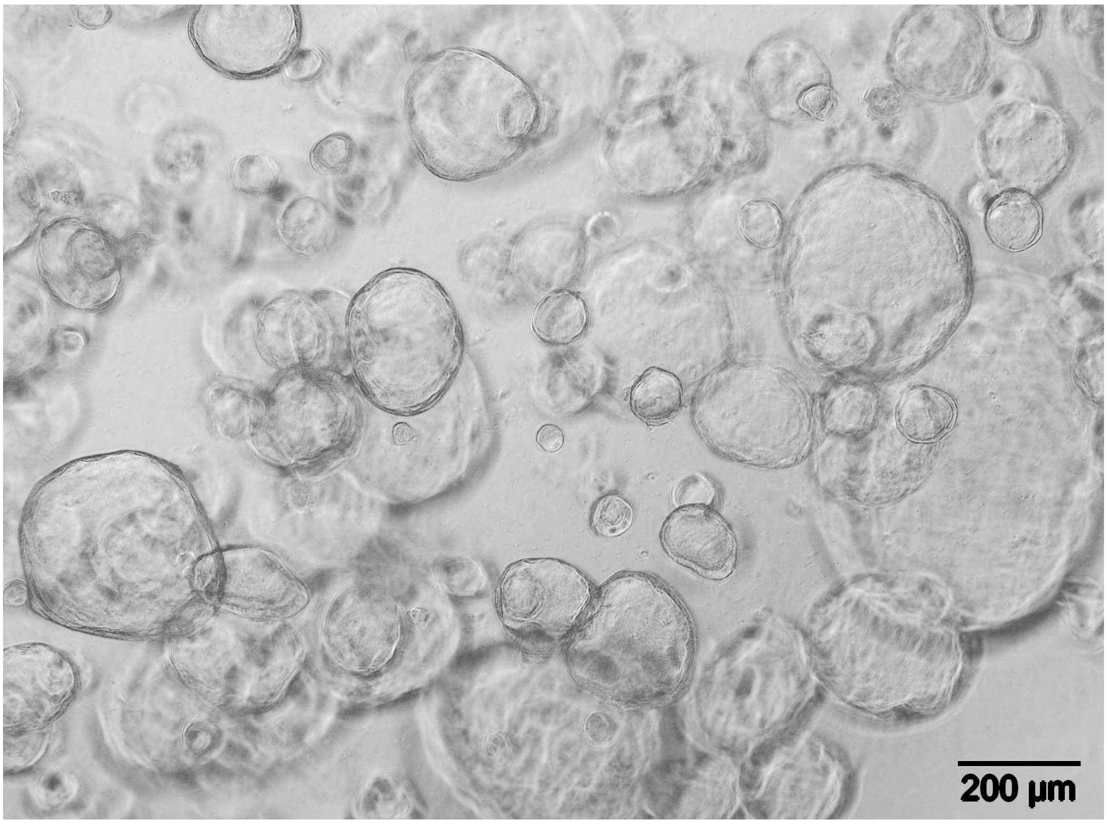

Stem-cell enriched organoid culture 5 days after passaging. Crypts have been obtained from the not inflamed sigmoid mucosa of a UC patient.

4. Organoid differentiation (day 8 to day 13)

56 Carefully aspirate the *organoid growth medium* from the culture generated in the previous section. Avoid detaching the Matrigel drops from the bottom of the wells.

57 Perform two washes of ≈ 250 µL/well for 10 min each with warm *basic medium* to remove the culture components by diffusion through the Matrigel dome. Meantime, prepare the *organoid differentiation medium*.

58 Add 250 µL of the *organoid differentiation medium* to each well.

59 After 2 days, add fresh *organoid differentiation medium* deprived of RSPO1. Terminal differentiation is usually achieved on day 5.

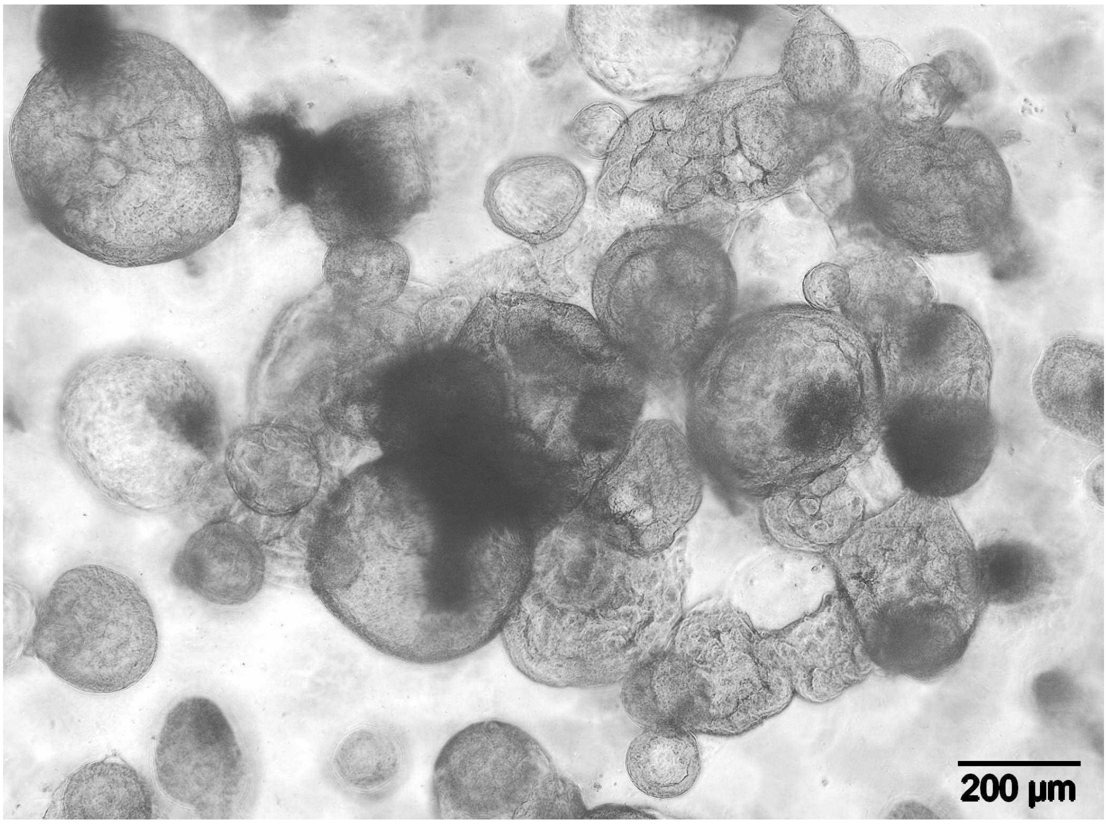

Organoid culture differentiated for 5 days. In this example crypts have been obtained from the non-inflamed sigmoid mucosa of a UC patient.
