# Supplementary figures and images for: Generation of human colon organoids from healthy and inflammatory bowel disease mucosa
Source: PLoS One. 2022 Oct 27;17(10):e0276195. doi: 10.1371/journal.pone.0276195 (PMC9612551; doi:10.1371/journal.pone.0276195)

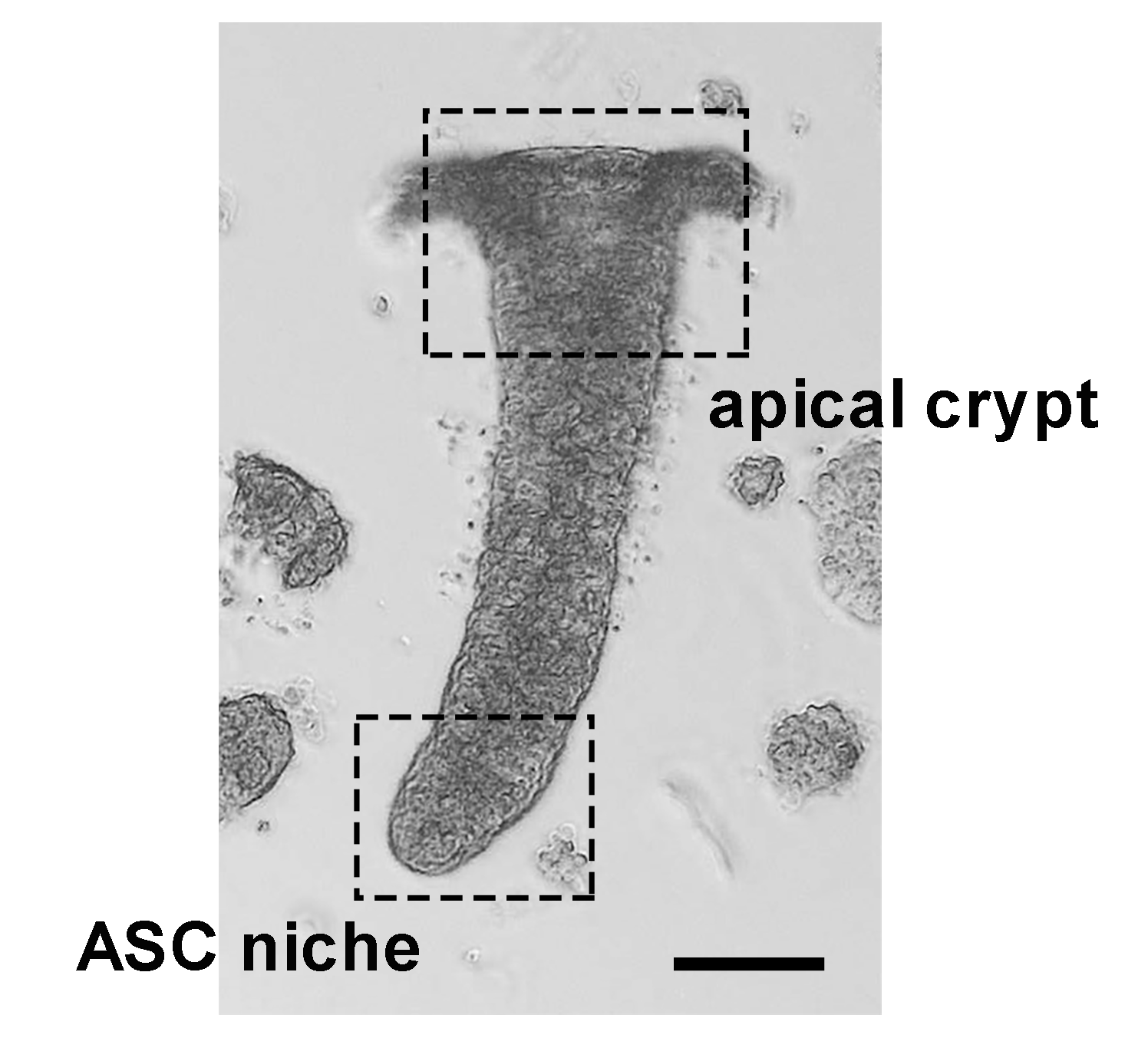

Supplement: S1 Fig — In this image, the bottom of the crypt containing the adult stem cell (ASC) niche and the apical crypt, where terminally differentiated cells are located, have been highlighted. This picture was taken from a supernatant enriched in crypts isolated from biopsies of the descending colon of a non-IBD donor. Scale bar: 100 μm. (TIF) [file pone.0276195.s002.tif]

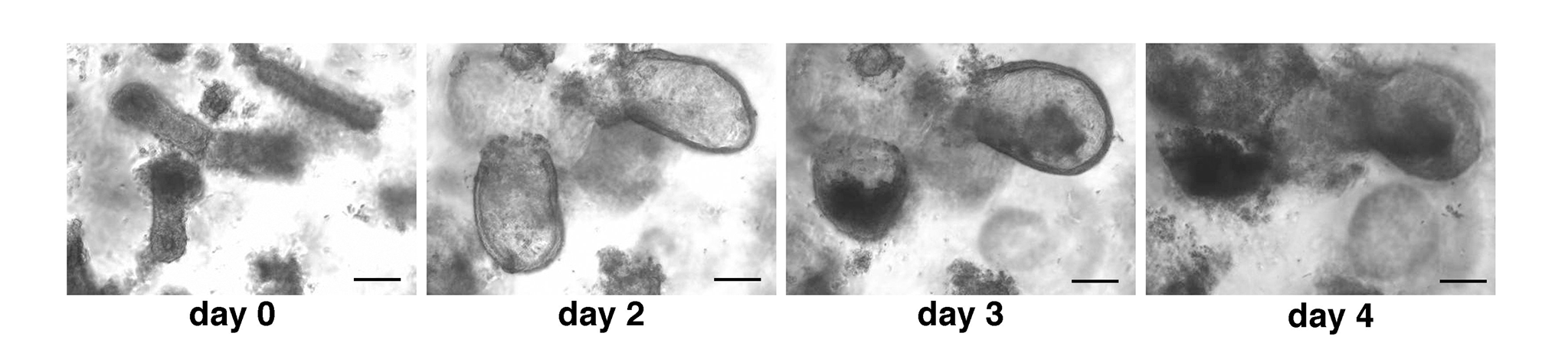

Supplement: S2 Fig — The viability of a crypt culture rapidly decreases over time due to the release of dead cells and debris into the lumen. This sample was obtained from the mildly inflamed sigmoid colon of an adult CD patient. Scale bar: 100 μm. (TIF) [file pone.0276195.s003.tif]

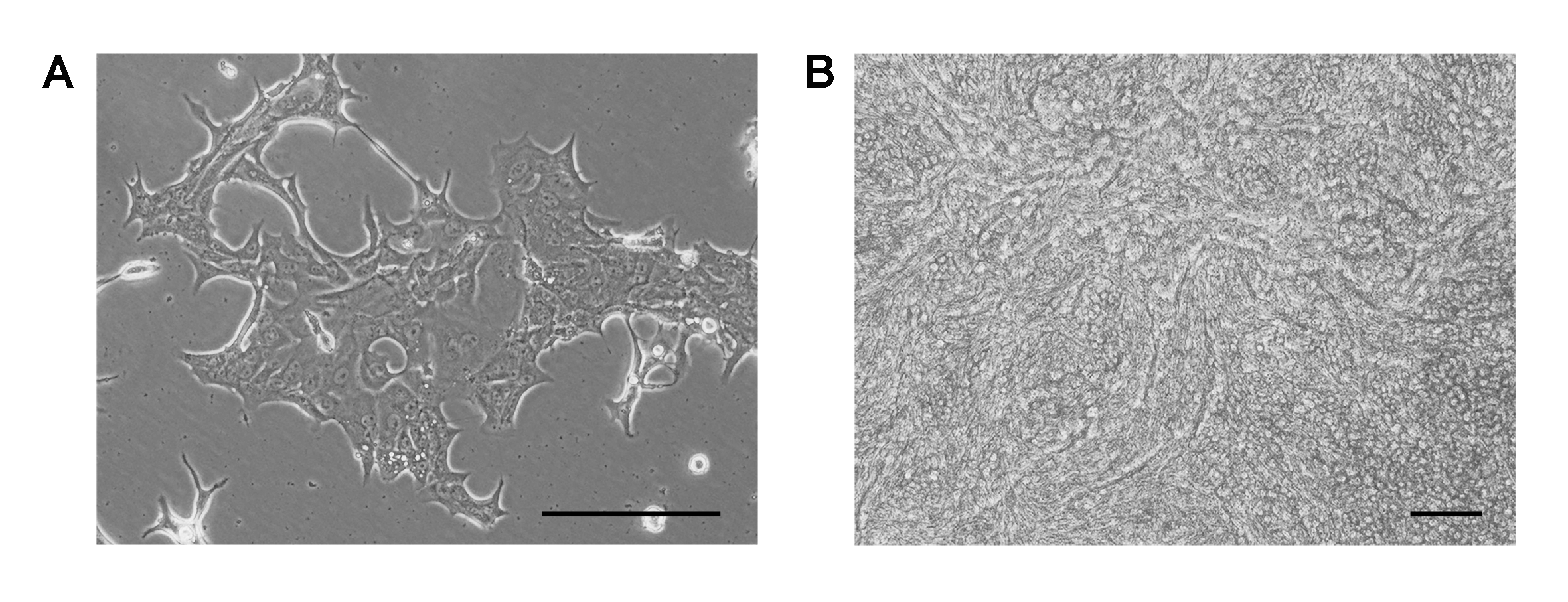

Supplement: S3 Fig — After organoid dissociation, epithelial single cells/small clusters of cells were counted using conventional methods and seeded on different supports. To promote cell adhesion and growth, a coating layer of diluted Matrigel was added. A) 2D epithelial culture at low confluence after seeding 5x10^4^ cells on a 48-well plate (pre-coated with 1:20 Matrigel). Image was taken 24h after cell seeding; B) Fully confluent 2D epithelial culture after seeding 5x10^4^ cells on a 0.33 cm2 Transwell insert (0.4 μm pore, pre-coated with 1:40 Matrigel). Image was taken 7 days after cell seeding. (TIF) [file pone.0276195.s004.tif]
