# Supplementary material for: Generation of human colon organoids from healthy and inflammatory bowel disease mucosa
Source: PLoS One. 2022 Oct 27;17(10):e0276195. doi: 10.1371/journal.pone.0276195 (PMC9612551; doi:10.1371/journal.pone.0276195)
Supplement: Attachment — Submitted filename: PlusOne Review Comment.docx [file pone.0276195.s005.docx]

On the Lab Protocol manuscript number; PONE-D-22-12935 entitled “Generation of human colon organoids from healthy and inflammatory bowel disease mucosa” the paper generates the following kinds of data;

1. Compared the organoids' growth and stability Ulcerative colitis and Crohn’s Disease are chronic inflammatory bowel diseases (IBD).
2. Described in detail a reproducible protocol to generate Matrigel-embedded epithelial organoids from ASCs of non-IBD and IBD donors using small colonic biopsies, including steps for its optimization.
3. Surgical biopsy samples were used.
4. Epithelial organoids can be expanded over several passages, thereby generating a large quantity of viable cells that can be used in multiple downstream analyses including genetic, transcriptional, proteomic and/or functional studies. In addition, 3D cultures generated using our protocol are suitable for the establishment of 2D cultures, which can model relevant cell-to-cell interactions that occur in IBD mucosa.

This paper is clearly written and well organized. the introduction and background are reasonable given the premise of the paper. figures and tables are comprehensive and helpful.

**Minor issues** **need to be dressed**

\# The author recommend using at least 5-6 donors when in vitro stimulation experiments are planned, and at least 12-15 donors per group when comparative analyses between cohorts of patients. In addition, as organoids derived from different segments of the intestinal tract show intrinsic differences (11, 33), also recommend, whenever possible, using biopsies from the same anatomical region to minimize this variability.

To modify the clinical applications of this method such as using internal control by collecting the samples from the patient’s colon healthy areas.

\# How many times successfully step 3 Step 3 can be repeated without changes in molecular features?
